# Single Photon Avalanche Diode Arrays for Time-Resolved Raman Spectroscopy

**DOI:** 10.3390/s21134287

**Published:** 2021-06-23

**Authors:** Francesca Madonini, Federica Villa

**Affiliations:** Dipartimento di Elettronica, Informazione e Bioingegneria, Politecnico di Milano, Via G. Ponzio 34/5, 20133 Milano, Italy; federica.villa@polimi.it

**Keywords:** time-resolved Raman spectroscopy, fluorescence suppression, single photon avalanche diode (SPAD), SPAD array, time gating, single photon counting (SPC), time-correlated single photon counting (TCSPC)

## Abstract

The detection of peaks shifts in Raman spectroscopy enables a fingerprint reconstruction to discriminate among molecules with neither labelling nor sample preparation. Time-resolved Raman spectroscopy is an effective technique to reject the strong fluorescence background that profits from the time scale difference in the two responses: Raman photons are scattered almost instantaneously while fluorescence shows a nanoseconds time constant decay. The combination of short laser pulses with time-gated detectors enables the collection of only those photons synchronous with the pulse, thus rejecting fluorescent ones. This review addresses time-gating issues from the sensor standpoint and identifies single photon avalanche diode (SPAD) arrays as the most suitable single-photon detectors to be rapidly and precisely time-gated without bulky, complex, or expensive setups. At first, we discuss the requirements for ideal Raman SPAD arrays, particularly focusing on the design guidelines for optimized on-chip processing electronics. Then we present some existing SPAD-based architectures, featuring specific operation modes which can be usefully exploited for Raman spectroscopy. Finally, we highlight key aspects for future ultrafast Raman platforms and highly integrated sensors capable of undistorted identification of Raman peaks across many pixels.

## 1. Introduction

Raman spectroscopy is a non-destructive technique which provides information on the physical and chemical structure of a material, based on the interaction of molecule vibrational modes with light. It is used in many scientific fields, such as medicine [1,2,3], geology and mineralogy [4,5], pharmaceuticals [6,7,8], and planetary science for the identification and characterization of unknown samples [9,10,11]. In this paragraph, we will present an overview of the physical phenomena at the basis of Raman effect, and then we will focus on Raman spectroscopy, discussing setup requirements and Raman-fluorescence trade-offs.

### 1.1. Raman Effect

The Raman effect or combinational scattering of light was discovered in 1928 by Sir C. V. Raman [12] and independently by G. Landsberg and L. Mandelstam [13]. It consists of an inelastic scattering of incident light photons that involves generating new frequencies during the light-matter interaction. These new frequencies are related to the vibrational and rotational modes of the analyte under observation [14]. Since these modes are a unique feature of each single molecule, Raman spectroscopy is a powerful tool to investigate molecular specificity and structure features. One of its greatest advantages is the label-free operation, which avoids any sample preparation and fluorescent marker introduction, making Raman one of the most versatile non-invasive real-time measurement technique.

**Figure 1:**
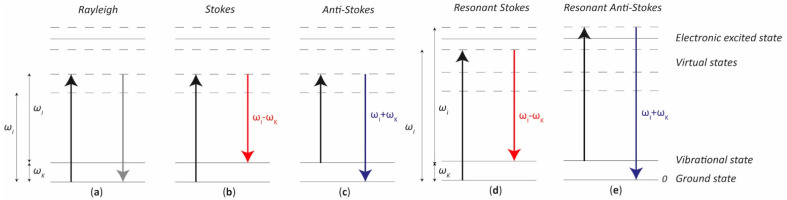
Simplified (**a**) Rayleigh, (**b**) non-resonant Stokes Raman, (**c**) non-resonant Anti-Stokes Raman, (**d**) resonant Stokes Raman, and (**e**) resonant anti-Stokes Raman scatterings. The arrows represent the electron jump from one state to the other: in black when the photon energy is absorbed, in grey when a Rayleigh scattering happens, in red and blue when Stokes and anti-Stokes Raman scattering respectively happen.

When the energy of an incident photon is not enough to fill the gap between two electronic energy levels, the molecule can be raised up to a virtual energy level, which is not a real state, but it assumes whatever energy the impinging photon has. Since the virtual state is not a stable energy level, it can be populated only during a transient, followed by a Raman or Rayleigh scattering. In such a case, we talk about non-resonant Raman scattering. With reference to Figure 1, given an incident monochromatic radiation of frequency ω_I_, if the molecule relaxes emitting a photon at the same frequency ω_I_, the scattering is of an elastic type, i.e., Rayleigh scattering (Figure 1a). Conversely, when the emitted photon frequency is lower or higher than the original one (i.e., ω_I_ ± ω_K_), an inelastic (Raman) scattering happens, referred to as Stokes (Figure 1b) and anti-Stokes (Figure 1c) Raman scattering, respectively.

One should note that ω_K_ is the resonance frequency of the considered vibrational energy level: in a Stokes scattering the molecule relaxes back to an excited vibrational level, whereas an anti-Stokes scattering happens when the molecule was originally on an excited vibrational level and, from a virtual state, it returns to the fundamental one.

Resonant Raman scattering happens when the excitation frequency ω_I_ is very close to the frequency of an electronic transition or, in other words, the virtual state energy level is very close to an electronics excited state, as shown in Figure 1d,e for the Stokes and anti-Stokes case, respectively. This feature makes the Raman process more likely to occur, resulting in a much stronger signal, thus greater sensitivity to detect lower substance concentrations or decrease exposure time.

### 1.2. Raman Spectra

**Figure 2:**
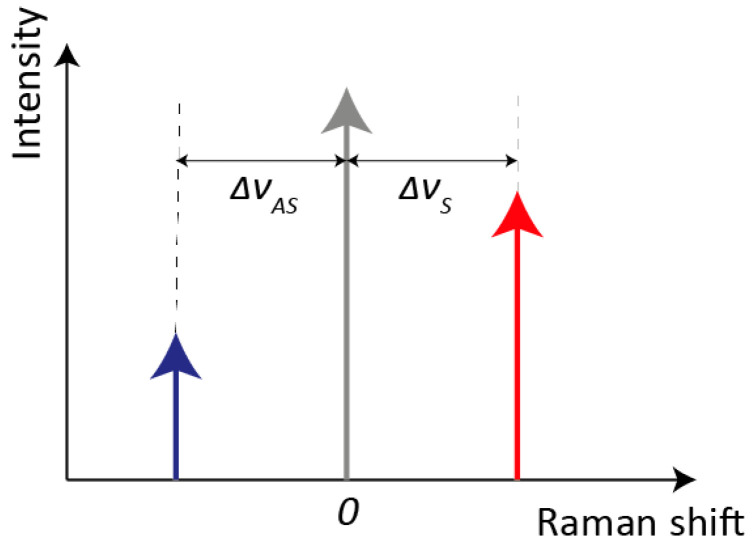
Schematic representation of Raman shifts for Stokes and anti-Stokes processes. The red and blue arrows represent, respectively, the intensities of Stokes and anti-Stokes Raman scattering, whereas the grey arrow represents the Rayleigh scattering (not in scale).

In spectroscopy, the commonly used unit is the wavenumber ν = 1/λ [cm^−1^], which allows the definition of the Raman shift Δν [cm^−1^] as the difference between the incident photon wavenumber ν_I_ and the outcoming photon wavenumber ν_O_. The Raman shift includes vibrational information related to each individual molecule, such as its structures and chemical bonds. Compared to Rayleigh scattering, Raman scattering is extremely weak, its intensity being 10^−6^ smaller than the Rayleigh one for pure liquids and down to 10^−12^ for powders [15]. As shown in Figure 2, if we refer to only one vibrational mode for sake of simplicity, Raman shifts for Stokes and anti-Stokes process (Δν_S_ and Δν_AS_, respectively) are equal in absolute value, since they are both created from the vibrational resonance frequency ω_K_ and the probe laser frequency ω_I_. Therefore, also the information contained within Stokes and anti-Stokes sides is the same. However, the intensity of Stokes is far greater than the one associated with anti-Stokes ones. Indeed, their intensity ratio is governed by the sample’s absolute temperature and the energy difference between ground and excited vibrational states. Since the majority of molecules are in the ground state at ambient temperature, the Stokes Raman lines are much more intense. An increase in temperature, which would cause an increase in the population of the excited vibrational states, is not a feasible solution for boosting anti-Stokes intensity because it could compromise the analyte structure.

Ambient light affects the quality of Raman signal, thus common straightforward solutions are to perform Raman measurements in a dark environment or place the sample in a stray-light sealed enclosure. A complete enclosure is difficult to be achieved with on-site outdoor measurements [16]. Rayleigh scattering also constitutes a strong background light overwhelming Raman bands due to its higher intensity; therefore, it must be rejected, for example, through an optical notch filter in applications exploiting both Stokes and anti-Stokes spectra [17]. Since filters with strong in-band attenuation and sharp edges should be employed in order not to compromise other spectral components, in conventional Raman spectroscopy, it is simpler to use a long-pass filter, cutting out also the anti-Stokes side (carrying the same information content of the Stokes one) while saving the higher intensity Stokes one.

Eventually, the Stokes Raman band is also overwhelmed by fluorescence, due to electrons excited to a higher electronic energy level that, after a relaxation time, return to their ground state through the emission of photons at a longer wavelength [18]. Fluorescence happens when the absorbed photon has enough energy to promote transitions between two allowed energy levels. Therefore, it is most likely to happen in resonant Raman spectroscopy, with source frequencies near to the ones corresponding to molecule electronic transitions. Due to its higher intensity, fluorescence signal typically overshadows Raman scattering, and it cannot be filtered without also eliminating Stokes band, since they are both located at longer wavelengths (i.e., lower energy).

### 1.3. Raman Spectrometer System

**Figure 3:**
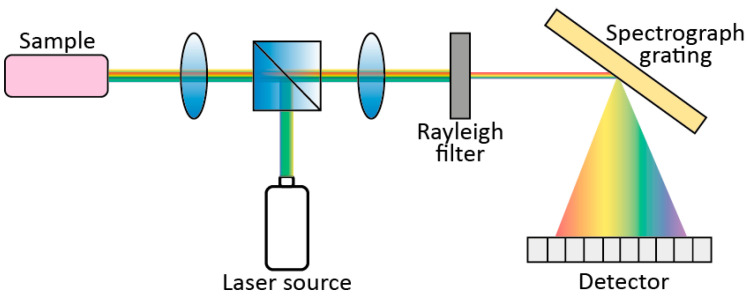
Typical components of a Raman spectrometer.

Figure 3 shows the five fundamental components of a dispersive Raman spectrometer: a laser excitation source; a light collection system for focusing light onto the sample and collecting the scattered light; an optical filter to cut out Rayleigh components; a diffraction element (e.g., a diffraction grating) to disperse the light into its spectral components; and a detector. The diffraction element selection is key for the final spectral resolution: full-scale range (FSR) and resolution depend on the spatial wavelength spread of the grating [19].

The laser frequency selection strictly relates to the desired fluorescent background rejection, as well as to the Raman signal intensity. In fact, Raman bands’ intensity is proportional to the inverse 4th power of wavelength [20]; therefore, shorter wavelength lasers give rise to stronger Raman intensities. However, the higher the photon energy, the higher the chance of exciting specific sets of fluorophores, which may contribute to fluorescence. Since both Raman and fluorescence increase at shorter stimulation wavelengths, a trade-off is needed. Laser wavelengths in the near Infra-Red (NIR) range (typically 785–1064 nm), where fluorescence is no longer a problem, often make the Raman intensity too low and thus the Raman-to-fluorescence signal disadvantageous. Furthermore, silicon detectors, which are less expensive than other III-V materials and can be integrated together with electronics in single-chip detection systems, have a low sensitivity in the NIR [21]. Using a UV (<400 nm) laser wavelength has a clear advantage in terms of Raman efficiency, but fluorescence is enhanced too. For deep UV (<260 nm), fluorescence stays in the visible range, thus not overlapping with Raman spectra [22]; however, optics, lasers, detectors, and high-resolution spectrographs are difficult to be designed and the related high photon energy risks to damage the sample. Furthermore, UV light would be absorbed just in a very thin surface layer, thus not allowing deep sample analysis. Eventually, techniques to eliminate or at least reduce fluorescence impact must be implemented.

Photobleaching permanently kills the fluorophore ability to fluoresce, due to photon-induced chemical damage and covalent bond modification. Such modification can be obtained by illuminating the specimen with the excitation laser over an extended period of time. Besides being slow, this process induces chemical and physical changes that cannot be tolerated by some materials (e.g., living cells). Thus, photobleaching is not a suitable answer to reduce fluorescence [23].

Eventually, different possible techniques to improve Raman signal compared to fluorescence have been reviewed in [24], where they are classified into time-domain, frequency-domain, wavelength-domain, and computational methods.

**Figure 4:**
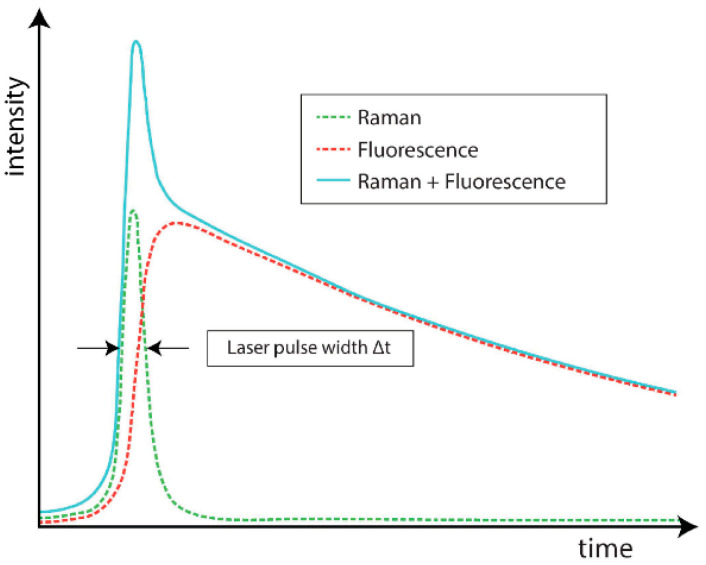
Raman and fluorescence signal time profiles [26]. Note that Raman intensity can be even much lower than the fluorescence one.

Among time-domain methods, which exploit the different time scales of Raman and fluorescence processes, an effective solution is the exploitation of Time-Gated (TG) detectors. Figure 4 shows a schematic time profile of a Raman band with a fluorescence background following the laser pulse. The fluorescence photon is emitted with an average characteristic delay, while Raman scattering is theoretically instantaneous, even if significant temporal broadening (even hundreds of picoseconds) can be measured from thick scattering samples [25]. The typical Raman time scales are in the sub-picoseconds to picoseconds range, whereas fluorescence ones are in the hundreds of picoseconds to nanoseconds range [26]. The limit in decreasing the laser pulse width to get an even shorter Raman signal is represented by the minimum laser power requested for Raman scattering, whose intensity is indeed proportional to the laser power. Reducing the laser pulse width requests increasing the laser peak power, which could lead to sample damage or alteration [27]. Using a TG photodetector, it is possible to record only the part of the emission pulse related to the Raman effect, while rejecting the fluorescence tail. Instead, the residual fluorescence (RFL) already emitted during the pulse, thus overlapping the Raman signal, cannot be rejected. Various algorithms can be exploited in post-processing to enhance the acquired signal accuracy, based on the identification of Raman spectra peaks, distinguishable only after TG operation [28,29,30,31].

**Figure 5:**
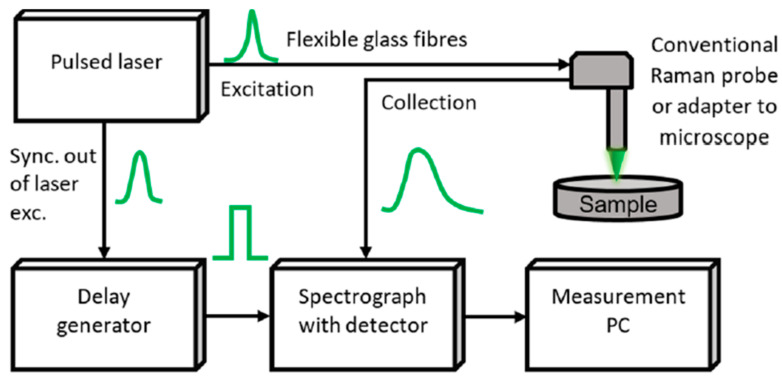
General block diagram of TG spectrometer, showing that the pulsed laser, through a delay generator, is used to trigger (i.e., to gate) the detector [32].

As highlighted in [32], a typical TG Raman setup includes a pulsed laser source with repetition rate in MHz range, picosecond-range pulse width, and suitable pulse energy. The latter must be enough to excite the sample spot, and just a minor fraction be used to synchronize the detector through a delay generator, matching the detection sequence delay, as shown in Figure 5.

## 2. Detectors

Photomultiplier tubes (PMTs) were the first detectors employed in sensitive Raman technique measurements [33]. They are based on photoelectric effect and secondary emission, and they are sensitive in the UV, visible, and NIR ranges of the electromagnetic spectrum. Nevertheless, they show several limitations. First, they are single-point detectors, meaning that they force each single spectral component to be scanned separately (i.e., the grating has to be moved and a new measurement acquired). Therefore, as the required spectral resolution increases, the acquisition time rises proportionally, and all experimental parameters have to remain constant during the scan. Furthermore, PMT devices cannot be electronically time-gated. Microchannel-plate photomultipliers (MCP-PMTs) solve spatial resolution issues since they include many channels. However, like PMTs they are not suitable for integration, and their photocathode can be damaged by prolonged exposure to light [34].

Charge-coupled devices (CCDs) have been the new generation of multichannel detection systems for Raman spectroscopy [35]. They are arrays of detectors storing a quantity of charge (proportional to the incoming radiation), which is then transferred from each element to the nearby one for readout [36]. By being an array, the whole spectrum can be acquired at once, with a clear advantage in terms of acquisition time. They ensure high sensitivity up to NIR [37], low noise related to dark current, even if they suffer from slow readout speed. Several research groups trigger CCDs by using Optical Kerr-gating [38], acting like a light shutter in front of the spectrometer entrance, or streak cameras [39]. However, such setups limit system portability due to the required bulky equipment. Intensified charge-coupled devices (ICCDs) provide a much more advanced solution for electronic gating (through the photocathode biasing) [40]. As a notable example of Raman measurements, ref. [41] reached a high fluorescence suppression with a state-of-the-art ICCD camera designed for fast gating at a repetition rate up to 110 MHz and a gate width shorter than 200 ps, time jitter lower than 20 ps, photocathode sensitivity in 400–900 nm range across 1376 × 1040 pixels, 10 frames/s readout, and 65% quantum efficiency. ICCDs main drawbacks are high power consumption (due to the high-voltage devices) often requiring Peltier cooling, photo-degradation (similar to PMTs), size, and cost. ICCD low frame rate is instead balanced by the ability to perform several counts per laser pulse, like any linear detector. Features, performances, and drawbacks of Kerr-gating and ICCDs are summarized in [42].

Single photon avalanche diodes (SPADs) are solid-state sensors based on reverse-biased p-n junctions where the electron-hole pair generated by the absorption of a single photon ignites an avalanche current build-up, easily sensed by the front-end electronics, which outputs a digital pulse. By switching the reverse voltage from slightly below to above the breakdown voltage and vice versa, SPADs can be easily time-gated on and off. CMOS SPADs are Si-made, and their major advantage is the easy integration with microelectronics since they can be made within the same fabrication processing. Regarding Raman spectroscopy, Si-SPAD main drawback is their low sensitivity in the NIR range, due to the relatively thin depletion layer of CMOS SPADs compared to thick-junction ones [43,44,45]. InGaAs/InP [46,47] or Ge [48,49], whose energy gaps are much lower than the Si one, are used to detect NIR photons, however they cannot be monolithically integrated with electronics and need to be cooled for reducing the intrinsic noise.

First demonstrations of TG Raman spectroscopy employing a single SPAD moved by a step motor with small steps to cover the full Raman shift range were reported in [50,51]. A strong fluorescence suppression was demonstrated with 300 ps time-gate windows. Then, the potential large-scale manufacturability of CMOS SPADs soon drove the development of SPAD arrays for TG Raman. Indeed, SPADs digital-like output pulses allow easy on-chip digital processing also for many-pixels arrays. In fact, SPAD arrays fulfil the desired spatial resolution, besides high sensitivity, limited power dissipation, no need for cooling over a wide temperature range, sub-ns time gating, and large-scale miniaturized chip formats.

A brief overview of SPAD-based detector for TG Raman together with a perspective on TG Raman historical development, applications, and extensions was presented in [32], while the purpose of the following paragraphs is to discuss the “ideal” features of SPAD arrays with integrated processing electronics for TG Raman spectroscopy and to illustrate some chip architectures already reported in literature. Compared to [52], which provides a complete overview on CMOS SPADs for biophotonics applications (including TG Raman spectroscopy), our review seeks to highlight design guidelines for optimized detectors targeted to innovative SPAD-based ultrafast and ultrasensitive Raman spectrometers.

## 3. SPAD Array Requirements for TG Raman Spectroscopy

**Figure 6:**
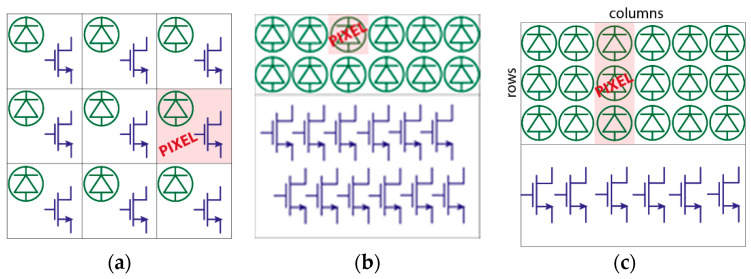
Example of (**a**) in-pixel and (**b**) out-of-pixel electronics for generic linear arrays, and (**c**) column pixels (associated to Raman lines) with out-of-pixel electronics for Raman linear arrays.

An array detector provides many pixels, laid out in a linear or two-dimensional format. Typically, there is no need for an array with a 1:1 (squared) aspect ratio in dispersive Raman spectroscopy, since the diffraction element spreads Raman wavelengths across a spectrum of lines. Hence, a linear array simplifies the whole system architecture because, by confining all detectors in a defined area, the electronics can be implemented aside, thus not impairing the Fill-Factor (FF), when considering standard planar technologies. FF is a fundamental figure of merit related to the SPAD active area, the pixel geometry, and the pixel array layout, and it is defined as the ratio between the detection active area and the total area illuminated by Raman light. Figure 6a,b underline the difference between a classical architecture with in-pixel electronic, usually employed in near 1:1 aspect-ratio arrays, and out-of-pixel electronics, more suitable for linear arrays. Out-of-pixel electronics also allows achieving a significantly smaller pixel pitch, thus increasing spatial resolution. As shown in Figure 6c, in a linear Raman array, the required number of pixels in the longer dimension, i.e., number of columns, depends on the number of spectral lines to be discriminated (i.e., wavenumber) and on the desired spectral resolution. These two parameters are specific for each Raman application and sample and depend on the optical system. The latter also defines the desired number of SPAD rows, that should match the transversal photon distribution given by the optical grating. In other words, more SPADs can be arranged in the same column pixels to increase the detected signal without losing in spectral resolution.

In-pixel electronics is no more an FF limitation when considering a 3D-stacked approach, where a top-tier includes SPADs fabricated in an optimized CMOS Image Sensor (CIS) process, while all processing electronics is implemented in a bottom-tier chip in a more scaled-down technology [53].

Discussing spatial resolution, the minimum distance between adjacent SPADs benefits from advanced isolation techniques, such as deep trench isolation (DTI) [54], which guarantees voltage isolation, parasitic reduction, and crosstalk minimization with narrow clearance among SPADs. Eventually, detector spatial resolution and pixel pitch directly translate into spectral resolution of Raman shifts.

Obviously, besides FF, high photon detection probability (PDP), defined as the ratio between detected and incident photons within the active area, is a key parameter to maximize photon detection. PDP is intrinsically related to the SPAD; FF depends on the array layout; the product of the two gives the overall photon detection efficiency (PDE). In Raman spectroscopy, the relative position of Raman bands with respect to the excitation frequency is independent of the excitation frequency itself. Therefore, the designer should choose an excitation frequency where the PDP is maximum. For further improving FF, hence PDP, a microlenses array (MLA) can be employed to precisely focus light onto the active areas.

The most relevant SPAD noise contribution is given by dark-count-rate (DCR) events, i.e., intrinsic avalanche generation rate of the detector in absence of illumination. A good quality fabrication technology, reducing lattice defects and minimizing generation-recombination centers [55], is essential to improve detector noise. In large-size arrays, it is common to find some “hot pixels”, which are pixels showing a DCR much higher than the median DCR of the array, usually related to local defects. Since their presence negatively impacts the imager performance, the array should embed a way to selectively disable them during an initial configuration. However, in TG SPAD arrays, the gate on/off duty-cycle helps decreasing the probability of detecting dark counts [51].

Combining a picosecond excitation pulse with a sub-nanosecond TG allows an effective main rejection of the broad unwelcome fluorescence. The rejection degree is roughly proportional to the ratio between fluorescence time constant and gate width [56], which should ideally match the laser pulse width to maximize the collection of Raman photons scattered during the pulse, but reject the delayed fluorescence. Indeed, different gate widths and positions determine signal-to-noise ratio (SNR) changes according to:

(1) $$SNR=\frac{N_{R}}{\sqrt{N_{R}+N_{F}+N_{DCR}}}$$

where *N*_*R*_, *N*_*F*_, and *N*_*DCR*_ are the numbers of detected Raman, fluorescent, and dark count photons, respectively, during time-gating [57].

In this regard, fast and precise detector gating capability is needed. Two different approaches are possible: “hard-gating” and “soft-gating”. In “hard-gating”, SPADs are disabled outside the gate window, by biasing them below the breakdown voltage through additional transistors in their front-end circuitry, that therefore should provide both a fast activation and deactivation of the SPAD [58]. In “soft-gating”, SPADs are not actively disabled, but the front-end’s digital output is masked to the subsequent processing electronics or, in other words, the counting or timing logic is disabled outside the gate window. In this case, rising and falling edges of the activation signal are only limited by the speed of the logic gates. In both approaches, the electronic blocks generating gate signals should be carefully designed to minimize time skews, which cause gate shifts and gate-width variations across array positions (i.e., wavelength points), becoming even larger when the number of SPADs in the array increases. This detrimental effect would give a different photon count at different spectral points even if the signal intensity remains the same [59]. To avoid distortion in the detected Raman spectrum, it is important that all pixels get activated in the same time interval, perfectly synchronized with Raman signal. [59] also computes signal-to-RMS-distortion ratio to define the quality of the spectra. Conversely, when fluorescence is rejected by post-processing time-to-digital converter (TDC) data, slower and less precise gate signals are needed if TDC resolution is enough to distinguish Raman and fluorescent photons. Also in this case, time skews related to TDC signals (e.g., delay lines and clocks) should be minimized, since they could affect the conversion precision. However, the TDC-based approach has the disadvantages of requiring a TDC per pixel, storing only one photon per pixel per time-frame, and slowing down the readout phase.

With high-performance timing electronics, the bottleneck could be set by the intrinsic SPAD performance, namely time-jitter and exponentially decaying tail [60]. Time-jitter refers to the statistical distribution of delays from the actual photon arrival to the actual detection time. Instead, the exponential tail is given by photons absorbed in the neutral region (instead of the high electric field depleted region of the SPAD junction), able to trigger avalanches after a not negligible delay from the actual photon absorption: it depends both on light wavelength and SPAD cross-section. Moreover, high excess bias is beneficial because it increases the avalanche current, which can be more precisely detected by the sensing electronics [61]. The obvious effect of these non-idealities is the time spreading of the SPAD ignition, thus the need of expanding the gate duration to match both laser width and SPAD jitter, thus increasing the detection of fluorescent photons present within the time gate and deteriorate the SNR according to (1).

When a photon is absorbed and the avalanche is triggered, the SPAD sensing and quenching circuit stops the current, preventing power dissipation, and restores the SPAD initial conditions after a given hold-off time [44]. The latter is necessary to release the charge trapped inside the junction, if any, that could retrigger the SPAD if brought above breakdown too soon, thus giving false events correlated to the primary ignition, called afterpulses [62]. Hold-off times in the 20–50 ns range are enough to limit afterpulsing to less than 1% in Si SPADs.

The gating frequency should be sufficiently high to repeat the laser excitation to collect a sufficient number of Raman photons within a reasonably short measurement time. At the same time, the repetition rate should be reduced to limit the overall system power consumption. The latter, indeed, is strictly related to the risk of overheating and consequently DCR increasing. If the chip temperature is kept limited without the need of a cooling system, the portability and compactness of the complete system is not compromised. Furthermore, TG inherently leads to significant dynamic power consumption, above all in the case of very fast on/off transitions applied to detectors with large capacitive loading. Therefore, particular care should be given to minimize cross-conduction currents (e.g., due to non-perfect synchronism between the signals that turn on and off the SPADs) and static power consumption due to leakage currents.

Finally, the array readout architecture plays an important role in achieving high frame-rate, hence short overall measurement time. For example, a double-buffered readout allows to perform a new acquisition while the previous data is being transferred to the on-chip electronics or to off-chip processing, virtually eliminating the typical dead time of global shutter.

## 4. Review of SPAD Arrays for Raman Spectroscopy

In this paragraph, we present an overview of the most interesting state-of-art SPAD arrays which have been on purpose-developed for Raman Spectroscopy or which present useful features for this field, even if originally aimed at different applications. Our goal is to describe the performance achieved in relation to the designers’ architectural choices.

### 4.1. 128 *×* 128 SPAD Camera

**Figure 7:**
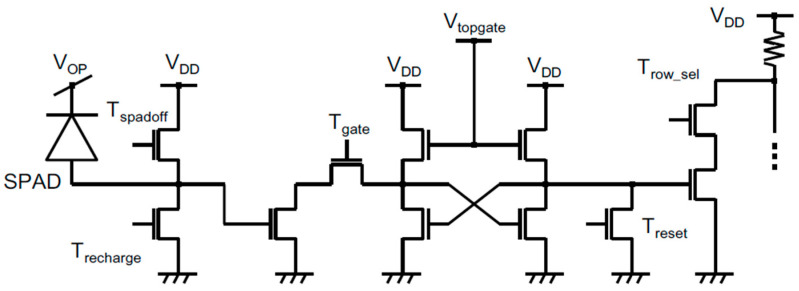
Pixel schematic diagram of the 128 × 128 time-gated SPAD camera in [63].

The time-gated 128 × 128 CMOS SPAD imager in [63] was originally designed for on-chip fluorescence detection and fluorescence lifetime imaging (FLIM) [64]. Fabricated in a 0.35 µm high-voltage standard CMOS technology, it features a total area of 20.5 mm^2^, 25 µm pixel pitch, and 4.5% FF. A single off-chip SPAD captures a small portion of the pulsed laser and activates an external delay line, which generates the timing signals fed to the array through an FPGA. On-chip delay cancellers and buffers are in charge of distributing those timing trigger signals throughout the whole array. As shown in Figure 7, each SPAD is enabled by a proper T_recharge_ signal, followed by a T_gate_ activation of the in-pixel 1-bit counter to store the Raman photon event, which is read out in a rolling shutter mode every 409 µs, through row/column decoders. Pixel data are on-chip accumulated and serialized. For PDE enhancement, the array is coupled with microlenses mounted on the sensor surface with a median concentration factor of 1.59. The minimum achievable time-gating is only 33 ns, due to initial choices related to the targeted FLIM applications.

### 4.2. 1024 *×* 8 SPAD Line Sensor

**Figure 8:**
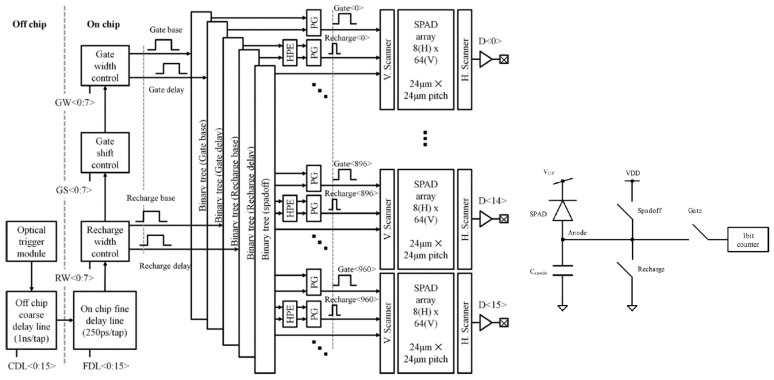
Block diagram of the 1024 × 8 Time-Gated SPAD Line Sensor in [66], with details on control signals used by each gated SPAD.

The sensor in [65,66] was designed in a 0.35 µm high-voltage CMOS technology specifically for TG Raman spectroscopy and Laser-Induced Breakdown Spectroscopy (LIBS) [67]. It includes 1024 × 8 square-shaped SPADs grouped in 16 blocks made of 24 × 8 SPADs each, for a total length of 24.7 mm, which matches a typical spectrometer output. Each pixel includes a SPAD, a 1-bit counter, a readout interface, and a 1-bit memory for disabling “hot-pixels”, altogether reaching a 44.3% FF with 24 µm pixel pitch. Pixels can be operated both in time-gated and free-running modes. As shown in Figure 8, the SPAD gate timing with respect to the laser pulse is controlled by an off-chip coarse delay line (1 ns/tap) and an on-chip fine delay line (250 ps/tap). The delayed trigger generates the control signals (“Spadoff” and “Recharge” to respectively bias the SPAD below and above breakdown, and “Gate” to enable event capture during an “on” state), propagated through balanced binary trees and Pulse Generators (PGs) in each row. Signal intensity is therefore retrieved by counting the number of photons at each pixel, i.e., at each spectral point. The minimization of the final propagation length and the attention paid to power supply stability ensure a time skew smaller than 100 ps across the entire array. The gate signal (minimum width of 0.7 ns) manages to turn on and off the SPAD with 250 ps accuracy and 32 ns scan range.

### 4.3. 128 *×* 1 Multiphase Time-Gated SPAD Line Detector

**Figure 9:**
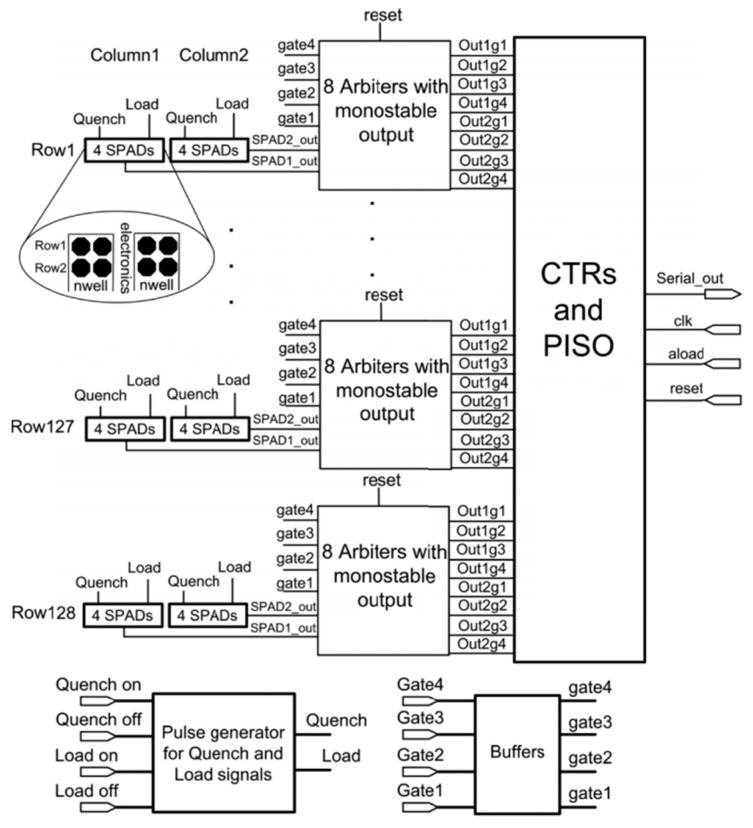
Block diagram of the multiphase time-gated SPAD line detector in [56].

The 0.35 µm CMOS SPAD-based line detector in [56] is designed to reject fluorescence and dark counts from Raman signal by counting photons during short time gates (t_1_, t_2_, t_3_, t_4_), which also allow estimating the fluorescence time constants (to furtherly remove it from the spectrum). This line detector has a size of 0.34 × 4.1 mm^2^, 33 µm pixel pitch, 23% FF, and it comprises 128 detector elements, each including 2 × 4 SPADs (i.e., 2 pixels made of 4 SPADs each). The double-column increases the detection area and gives the possibility to exclude noisy detectors. As shown in Figure 9, each column has common “Quench” and “Load” signals to respectively turn off and on the SPADs. These signals come from an on-chip pulse generator, whose inputs are triggered by the laser pulse and can be off-chip adjusted. The laser pulse also triggers gate signals (gate_1_, gate_2_, gate_3_, gate_4_) with durations of 100, 200, 300 and 800 ps, respectively, by exploiting an off-chip delay circuit.

Arbiters recognize if a photon is detected between “Load” and every gate signal, producing corresponding output pulses. A counter block (“CTRs and PISO”) includes 3-bit counters for each output to track the number of photons detected by each column within time gates. Even if overlapping time gates give redundant information, the architecture was chosen because of its simplicity. A parallel-input-serial-output (PISO) register transfers the counter data to the external FPGA without stopping photon detection during readout. The maximum measured variation of time gates was ±35 ps across the entire array, thus limiting the system accuracy.

### 4.4. 256 *×* 2 TRFS Line Sensor

The SPAD line sensor in [68], which is an upgrade of a previous work in time-resolved CMOS SPAD line sensors [69], is mainly designed for time-resolved fluorescence spectra (TRFS) [70] with the aim of speeding up time-correlated single photon counting (TCSPC) acquisitions [71]. However, it can also work in single photon counting (SPC) and center-of-mass (CMM) modes. CMM provides on-chip lifetime estimation of fluorescence decays. The array is fabricated in a 130 nm CMOS technology and includes two rows of 256 pixels, each composed of four SPADs optimized for the blue spectral range and four SPADs optimized for the red spectral range, with 43.7% FF and 23.78 µm pixel pitch. It integrates 256 × 26-bit TDC channels, acting as a TCSPC TDC counter in TCSPC mode and as a photon counter in SPC mode. The same circuit controls time-gating behaviour in both TCSPC and SPC modes.

**Figure 10:**
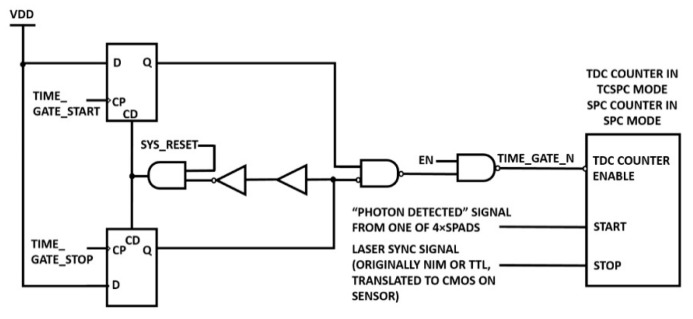
Time-gating circuit of the 256 × 2 TRFS Line Sensor in [68].

Instead of time-gating SPADs by modulating their excess bias (causing electrical transients on the SPAD high voltage supply), the implemented time-gating architecture works by digitally discarding at the SPAD output stage those photons detected in the off region. In other words, SPADs are kept on all the time. As shown in Figure 10, TIME_GATE_START and TIME_GATE_STOP are clock inputs of two D-flip-flops defining the time window in which the TDC is enabled. In TCSPC, the TDC is activated by a photon detection and stopped by the laser pulse. Therefore, TIME_GATE_STOP is generated from the laser pulse through an on-chip delay. In SPC, instead, the time-gating window defines the region in which photon counting is performed. To focus on time events of interest, the user can change the position and width of the time-gate window through a 128 step on-chip delay line separate from the TDC circuits. The shortest time-gate window was measured to be 1.4 ns. Parallel access to data lines coming from pixels was designed to increase readout speed up to 19,000 lines/s. Main reported TDC issues in TCSPC are coarse time resolution (0.43 ns) and coarse jitter (>1ns). Due to firmware limitations, the minimum TCSPC exposure time is 8.3 µs. Among many applications, this SPAD-based line sensor was exploited in SPC mode in a low-resolution Raman spectroscopy (LRSS) experiment with a broad peak, where 5.6 ns on time-gate was employed.

### 4.5. 256 LinoSPAD Camera

**Figure 11:**
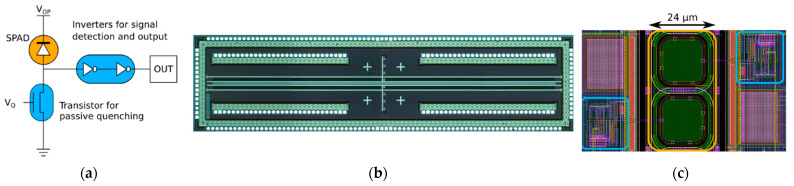
(**a**) Single-pixel schematics, (**b**) micrograph of LinoSPAD, and (**c**) two pixels layout [72].

The LinoSPAD camera system in [72] combines a linear 256-pixel CMOS SPAD sensor with 64 FPGA-based TDC modules to separately optimize SPAD performance and time-stamping processing. The SPAD chip fabricated in a 0.35 µm high-voltage CMOS process consists of a row of 256 pixels with integrated passive quenching (i.e., quenching transistor and two inverters, as shown in Figure 11a. A high FF value of 40% is reached with a pixel pitch of 24 µm. Through a PCB carrier, each pixel digital output is connected to a Spartan 6 FPGA fabricated in a 45 nm process, easily adapting to the most various applications. Each FPGA-TDC can process over 80 million photon detections per second with an average resolution of 50 ps. To cope with the large number of I/O pads in a limited area of 6.8 mm × 1.7 mm, additional pads were also placed inside the traditional pad ring of 192 elements, as shown in Figure 11b.

### 4.6. 16 *×* 256 TDC-Based SPAD Line Detector

**Figure 12:**
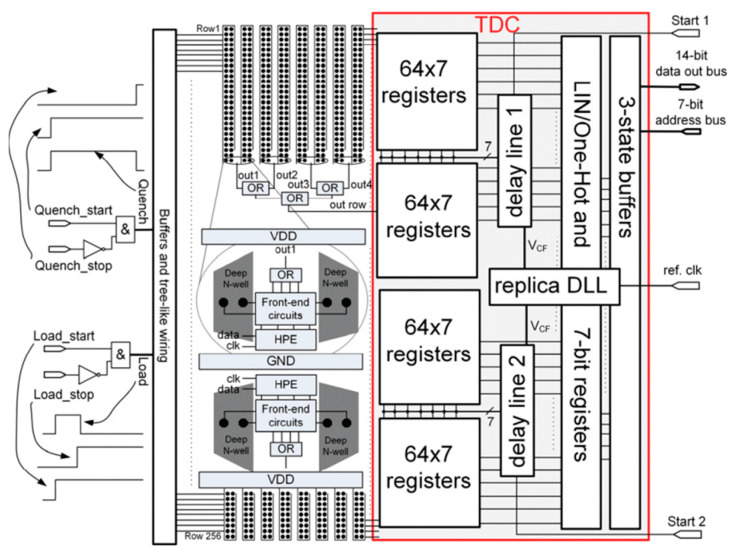
Block diagram of the time-gated 16 × 256 SPAD line sensor with on-chip 256-channel TDC [73].

The 16 × 256 SPAD array in [73], manufactured in a 0.35 µm high-voltage CMOS technology, embeds an integrated 3-bit TDC with 256 channels. With a TDC approach, photon arrival times at every spectral point are recorded with high-enough time resolution, so that Raman photons can be distinguished from fluorescence ones in post-processing. In principle, the TDC LSB should match the laser pulse width, and all Raman photons could be detected in the first TDC bin. However, TDC FSR is usually expanded to cover the entire Raman signal even in presence of time-skews. Moreover, an FSR expansion allows measuring part of the fluorescence background to be used in post-processing to minimize the RFL. A fast optical detector is used to capture the laser pulse and consequently activate the SPADs and start the TDC (after a delay). As shown in Figure 12, the 16 SPADs in each row are placed in couples in a way that the relative electronics are enclosed between two of them. Such electronics include: SPAD front-ends to enable/disable each detector and sense the avalanche triggered by a photon; hot-pixel elimination circuit; OR gates to connect each row to an input of a register. The enable and disable signals (“Load” and “Quench”, respectively) are generated by four off-chip rising edges, whose relative delays are adjustable with a resolution of 250 ps. A tree-like wiring and buffering system are used to distribute them to all SPADs maintaining simultaneous loading and quenching. OR gates also are carefully connected to equalize timing skews between SPADs in the same row (i.e., same spectral point).

Each row has its own TDC channel, which operates by storing the state of the 7 TDC phases in a 7-bit register, in response to a detected photon. The 7-phase delay line is split into two identical lines, each delivering phases to half of the rows, to minimize phase skews. TDC variations in temperature and supply voltage are compensated with a replica delay-locked loop, which is delay-locked to an off-chip reference clock. Changing the reference clock frequency between 50–100 MHz makes it possible to adjust TDC resolution in the 50–100 ps range and dynamic range between 350–700 ps. Due to timing skews higher than expected, the 50 ps resolution could not be used, and the 100 ps one brought to a maximum skew of ±75 ps. With a 14-bit parallel output bus, a reading time of 2.5 µs is reached (i.e., 400 kframes/s). The chip size is 9 mm × 3 mm, the FF along the spectral axis 26%, and the pixel pitch 35 µm.

A previous work [74] obtained a 78 ps resolution, but only in a limited measurement range of 300 ps. It consists of a time-gated 4 × 128 SPAD array based on a 512-channels TDC, with a chip architecture similar to the 16 × 256 TDC-based SPAD Line Detector [73]. The main difference is a high resolution (78 ps) designed only for the first four TDC bins to accurately time-stamp Raman photons, while the last four bins were increased in width for more-relaxed fluorescence measurement at the end of the range.

### 4.7. 512 *×* 16 SPAD Line Sensor with Per-Pixel Histogramming TDC

The line sensor designed for time-resolved multispectral imaging in [75] can operate in SPC mode (102.1 giga-events/s), TCSPC mode (192.4 million-events/s), and on-chip histogramming mode (16.5 giga-events/s). Fabricated in a 130 nm CMOS imaging process, it includes two SPAD line arrays, one (49.31% FF) optimized for detection in the blue-green (450–550 nm) spectral region and the other (15.75%) optimized for the red-near-infrared (600–900 nm) wavelengths.

**Figure 13:**
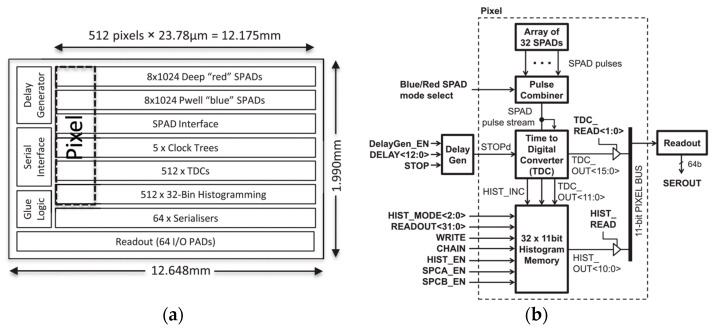
(**a**) Sensor and (**b**) pixel block diagrams of the 512 × 16 SPAD Line Sensor [75].

Each pixel has 2 × 8 blue SPADs and 2 × 8 red SPADs. Multiple smaller SPADs have been chosen to increase sensitivity while having lower time jitter, crosstalk, and DCR than a single larger detector. Pixel pitch measures 23.78 µm. Pixels also include SPAD quenching/sensing circuits, single-bit memory cells for hot-pixel disabling, a 16-bit TDC, and an 11-bit/bin histogram memory. As shown in Figure 13a, the sensor also features five clock trees (to distribute laser-synchronized signal for TDCs and time-gates), 64 serializers, a readout logic, a delay generator, and a serial interface. Figure 13b shows the pixel block diagram. Only blue SPADs are gated on/off by rapidly changing their bias voltage. “Pulse combiner” selects only one SPAD variant providing short pulses per photon count. The TDC is based on a gated ring-oscillator operated in reversed start-stop configuration (i.e., started by a SPAD pulse and stopped by the laser signal) to minimize power consumption, and it is compensated for process, voltage, and temperature (PVT) variations.

The on-chip delay generator allows synchronization with the target signal and is implemented with the same gated ring-oscillator. TDCs are activated in TCSPC and histogramming modes. In the latter, more than one event can be captured in each exposure time, increasing the pixel dynamic range. Moreover, since only final histograms are read instead of all photon events, I/O power consumption is reduced. Adjustable TDC bin widths from 51.20 ps to 6.55 ns allow capturing fluorescent decay times as well as short time scale Raman signals. This feature was demonstrated in [76], where time-resolved separation of fluorescence background and Raman scattering of liver tissue for transplant was achieved only based on pixel time dataset. Eventually, each serializer feeds an output pad and is shared by eight pixels, allowing a full-chip readout in 2.2 μs with a 40-MHz clock. The sensor size is 12.6 × 2.0 mm^2^.

### 4.8. 128 *×* 1 Time-Gated SPAD Array

**Figure 14:**
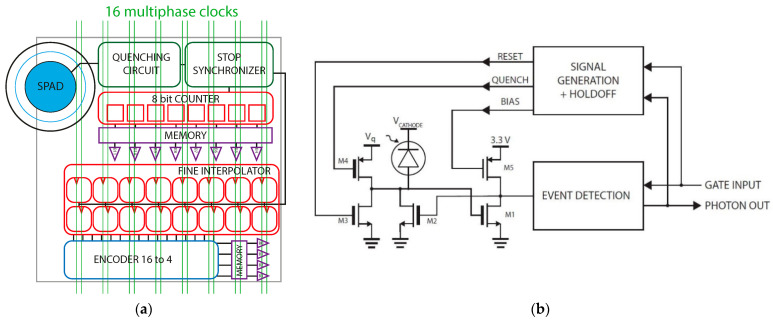
(**a**) Smart-pixel block diagram and (**b**) SPAD front-end circuitry for active gating [78].

The 128 × 1 SPAD array presented in [77,78] designed for various time-resolved applications, such as 3D ranging [79], Diffuse Optical Tomography (DOT) [80], quantum experiments [81], FLIM [64], and Raman spectroscopy, performs both photon-counting 2D “intensity” images and photon timing 3D (time-resolved) maps. It is fabricated in a 0.35 µm high-voltage CMOS technology and, as shown in Figure 14a, each pixel includes a SPAD with its front-end quenching and sensing circuit, an 8-bit counter, a 12-bit TDC, and internal memories. The TDC is based on a 16-phases clock interpolation scheme, with separate START and STOP 4-bit interpolators to adopt the sliding scale technique for linearity improvement [82]. The START interpolator is shared by the whole array, while each pixel includes the STOP interpolator, triggered by a photon detection, since TDCs work with START and STOP in direct configuration. The 8-bit counter for photon-counting mode can also be exploited to extend the TDC FSR up to 1 µs. A 260 ps resolution is reached with particular attention in the design of the clock generation and distribution circuits. As shown in Figure 14b, the SPAD front-end circuit can also actively gate on and off the detectors by bringing their bias voltage above or below breakdown. In particular, transistors M1 and M2 sense the avalanche, “event detection” block masks spurious events synchronous with the SPAD disabling, “signal generation + hold-off” block ensures the correct hold-off time after each triggering event, and drives M3 and, through a level shifter, M4, which, respectively, reset and disable the SPAD when operated in gated mode. This active gating performs subnanosecond transitions (780 ps), allowing efficient time-domain filtering of incoming light, such as selectively avoiding “early” photons, which would saturate the SPADs in applications where only “late” photons are useful (e.g., deep biological tissue’s layers analysis). Falling edges resulted faster than rising ones, since they are given by the masking operation of the “event detection” block, whereas rising edges represent the actual excess bias provided to the SPADs.

With 75 µm pixel pitch and 30 µm SPAD diameter, the FF is 12.5%, which can be improved with an MLA, up to the theoretical limit of about 78% [83]. The array features a double-buffering and global-shutter operation, meaning that the readout is concurrent with the acquisition of the following frame (5 µs minimum duration), reducing the dead-time.

## 5. Discussion

A complete table comparing state-of-art SPAD arrays for different Raman spectroscopy experimental setups in terms of technology, pixel number, pixel pitch, FF, PDE, and DCR is reported in [42]. Our review instead aims at comparing the architectural choices of existing SPAD arrays, from the microelectronics design point of view, in order to provide guidelines for an improved next-generation of Raman single-photon cameras.

Not all SPAD arrays presented in Section 4 were designed for TG Raman spectroscopy, even if they have been used for it. Some of them were conceived for FLIM, which aims at reconstructing lifetime decays of fluorescent molecules spanning from nanoseconds to milliseconds range. Among them, there are the 128 × 128 SPAD Camera [63], the 256 × 2 TRFS Line Sensor [68], and the 512 × 16 SPAD Line Sensor with per-pixel Histogramming TDC [75]. Furthermore, the latter features a time-zooming capability that reaches fine time bins suitable also for Raman spectroscopy as well as other multispectral imaging techniques. The 128 × 1 time-gated SPAD array [78] is a multi-purpose detector for time-resolved imaging applications. Conversely, the 1024 × 8 SPAD Line Sensor [66], the 128 × 1 Multiphase time-gated SPAD Line Detector [56], and the 16 × 256 TDC-based SPAD Line Detector [73] were developed on purpose for TG Raman spectroscopy.

The 128 × 128 SPAD Camera [63], the 1024 × 8 SPAD Line Sensor [66], and the 128 × 1 Multiphase time-gated SPAD Line Detector [56] are based on an SPC approach. They include no TDC, and they reconstruct fast signals by counting photons at every spectral position during limited time windows. The 256 × 2 TRFS Line Sensor [68] is based on a 256-channel 26-bit TDC performing either TCSPC and SPC (in TCSPC, TDC resolution is 0.43 ns with > 1 ns jitter). Also the 16 × 256 TDC-based SPAD Line Detector [73] features a 256-channel 3-bit TDC, which time-stamps photon arrivals with 100 ps resolution and 75 ps time skew. With a chip architecture very similar to the 16 × 256 TDC-based SPAD Line Detector [73], the work in [74] adopted a TDC with high-resolution (78 ps) LSB only for the first four bins to accurately record Raman photons, and a more relaxed resolution for the last four bins to detect the remaining emission (mostly related to delayed fluorescence). Per-pixel TDCs were proposed in the 512 × 16 SPAD Line Sensor with per-pixel Histogramming TDC [75], where the 16-bit 51.20 ps resolution TDCs can also perform histogramming. Per-pixel 12-bit 260 ps resolution TDCs are found in the 128 × 1 time-gated SPAD array [78], which can also work in SPC thanks to in-pixel 8-bit counters. An off-chip timing approach is instead proposed by the 256 LinoSPAD camera [72], where the 256 high FF pixel digital outputs are transferred to an external FPGA containing a TDC. With an average resolution of 50 ps, this off-chip timing concept is suitable for Raman spectroscopy too, though system portability and compactness are compromised. SPC approaches are simpler and less power-demanding than TDC-based ones, thus resulting very suitable for miniaturized TG Raman systems. However, TDC-based arrays enable further possibilities, for example, to measure the background fluorescence decay constant and consequently estimate the fluorescence photon counts overlapped to Raman ones.

Not all reviewed arrays perform the same gating method. The 128 × 128 SPAD camera [63] and the 1024 × 8 SPAD line sensor [66] act on the synchronous activation with the laser pulse of both SPADs (by changing their bias voltage) and in-pixel 1-bit counter for more precise time filtering. Also in the 128 × 1 multiphase time-gated SPAD line detector [56], SPADs are charged synchronously with the laser pulse, and a second time filtering is implemented by four different-duration gate signals, each one activating a counter that keeps track of the number of photons detected within the corresponding time window. In the 16 × 256 TDC-based SPAD line detector [73] and the 512 × 16 SPAD line sensor with per-pixel histogramming TDC [75], gating signals are used both for SPADs and TDCs activation. In the 128 × 1 time-gated SPAD array [78], active gating is performed by the SPAD front-end circuits, which also include a logic block to mask spurious events synchronous with the SPAD disabling. On the contrary, SPAD activation/deactivation is avoided in the 256 × 2 TRFS line sensor [68], where gating signals are only used to enable the TDC. As a general remark, counters or TDCs gating is advantageous from the power dissipation point of view, but SPAD gating is favourable when using a high repetition rate laser source. Indeed, it avoids the SPAD being blinded due to a former fluorescence trigger just before the next laser pulse.

As described in Section 1.2, gating signal widths in the sub-nanoseconds range should be pursued to discriminate Raman peaks from fluorescence background. In fact, the minimum achieved gating times were 33 ns in the 128 × 128 SPAD camera [63], 0.7 ns with 250 ps accuracy in the 1024 × 8 SPAD line sensor [66], 100 ps gate with 35 ps accuracy in the 128 × 1 multiphase time-gated SPAD line detector [56], 1.4 ns in the 256 × 2 TRFS line sensor [68]. In the 16 × 256 TDC-based SPAD line detector [73], gating signals are generated by an off-chip delay line with 250 ps resolution, and in the 512 × 16 SPAD line sensor with per-pixel histogramming TDC [75], by an on-chip delay generator with 51.20 ps resolution.

Aiming at very compact Raman detection systems, external delay lines for synchronizing gating signals with the laser pulse result bulkier than on-chip ones. The 128 × 128 SPAD camera [63], the 128 × 1 multiphase time-gated SPAD line detector [56], the 256 × 2 TRFS line sensor [68], and the 16 × 256 TDC-based SPAD line detector [73] have off-chip delay lines. The 1024 × 8 SPAD line sensor [66] implements a hybrid approach with off-chip coarse delay line and on-chip fine one. While only the 512 × 16 SPAD line sensor with per-pixel histogramming TDC [75] embeds an on-chip delay generator based on the same gated ring-oscillator used for TDCs.

Skew minimization measures are implemented in all reviewed arrays to avoid Raman spectra distortion due to gate signals or TDC signals mismatches throughout the array. To name a few: on-chip delay cancellers in the 128 × 128 SPAD camera [63], minimization of final propagation length and attention to power supply stability in the 1024 × 8 SPAD line sensor [66], distribution trees for laser-synchronized TDCs in the 512 × 16 SPAD line sensor with per-pixel histogramming TDC [75]. And again, tree-like wiring for simultaneous loading and quenching signals, path equalization between same row SPADs, and TDC delay line split in the 16 × 256 TDC-based SPAD line detector [73].

Concerning the array readout operation, the 128 × 128 SPAD camera [63] features a rolling shutter mode with 409 µs frame time. The 128 × 1 Multiphase time-gated SPAD line detector [56] performs 13 kframe/s without stopping the acquisition, as well as the 16 × 256 TDC-based SPAD line detector [73] with 400 kframe/s, the 512 × 16 SPAD line sensor with per-pixel histogramming TDC [75] with approximately 450 kframe/s, and the 128 × 1 time-gated SPAD array [78] with 200 kframe/s. The 256 × 2 TRFS line sensor [68] features parallelized access to pixel data to increase the readout speed to 19,000 lines/s. A no dead-time readout with continuous acquisition is fundamental to speed up Raman spectra measurements. Moreover, as a future trend, additional on-chip processing electronics could be included, e.g., to provide the address of pixels detecting higher intensity Raman peaks, so to speed up molecule identification.

After having described the architectures behind the reviewed arrays, a discussion on their power consumption can be made. A numerical comparison is not fair due to the different reported operating conditions (i.e., system clock, laser repetition rate, supply voltage). Qualitatively, it is worth mentioning two opposite approaches. The 256 LinoSPAD camera [72] with completely external processing electronics showed few mW power consumption in the dark and approximately 2 W for maximum activity, mainly related to many switching output pads (256 such as the pixel number), not to mention the FPGA-related power consumption. While the 512 × 16 SPAD line sensor with per-pixel histogramming TDC [75] features a histogramming mode to zoom on the peak of the fluorescence lifetime decay, where only final histograms are read instead of all photon events, thus reducing power consumption down to 168.10 mW. In general terms, to minimize power dissipation, TDCs, when present, are operated in reversed start-stop configuration (i.e., only started when a SPAD detects a photon and stopped by the next laser signal) in order not to start TDCs and counters when no photon detection takes place. Only the 128 × 1 time-gated SPAD array [78] works with direct start-stop configuration, to favour those applications with low frequency, not stable, or not externally triggered laser, which would require the introduction of long delays to provide a global synchronization at the end of the measurement cycle.

Table 1 summarizes the main architectural choices of SPAD arrays presented in Section 4 in terms of detection method (SPC or TCSPC), gating mode (“hard-gating”, i.e., activation/deactivation of SPADs, or “soft-gating”, i.e., activation/deactivation of processing electronics), delay line implementation (off-chip or on-chip), and readout mode (rolling shutter or global shutter).

## 6. Conclusions

For almost a century, the measurement of Raman shifts allowed molecule identification with neither sample preparation nor labelling in various fields, ranging from medicine and biology, to mineralogy, archaeology, and space science. Still today, despite being a well-established method, retrieving Raman spectra with portable and cheap devices in presence of a strong fluorescence background is still an open issue. The fluorescence reduction technique reviewed in this paper is the time-gated Raman spectroscopy: TG detectors exploit the different time behaviour of Raman (sub-picoseconds to picoseconds time scales) compared to fluorescence (hundreds of picoseconds to nanoseconds range) emissions after each laser excitation pulse. In a typical setup, the laser trigger output feeds a delay generator to subsequently enable the detector, so to record Raman photons only. Actually, also the residual fluorescence emitted during the pulse is recorded.

Among other single-photon detectors, SPAD arrays show huge advantages in terms of sensitivity, immunity to readout noise, power dissipation, possibility of being integrated together with on-chip processing electronics, and, above all, suitability to be easily time-gated. The requirements for an optimal TG Raman SPAD array have been discussed. Considering a future trend towards fast, portable, and cheap TG Raman systems, SPAD array designs should aim at increasing the gating frequency, enhancing the time resolution to perfectly match the laser pulse width with minimum skew-related distortions, reducing power dissipation, operating at room-temperature with no need for cooling, and maximizing frame-rate to speed up measurements.

Many state-of-art SPAD array architectures have been reviewed. Given the linear spread of Raman spectrum at the system’s optical output, almost all reviewed arrays have a linear format with out-of-pixel electronics for FF maximization and pixel pitch minimization. The main basis for comparison is gating capability. The bottleneck in achieving short gate-on width, thus more efficient fluorescence rejection, is set by the electronics performance and not by the SPAD intrinsic timing performance. When Raman signal reconstruction is accomplished in SPC mode, ultimate limits stand in the resolution and precision of the delay unit generating gating signals and in the capability of routing them throughout the array while maintaining sharp edges and zero skews. For future improvements, on-chip delay lines allow better results than commercial off-chip ones in terms of stability, time resolution, and linearity. When TCSPC is adopted and fluorescence is rejected by post-processing TDC data, ultimate limit become TDC linearity. However, when compared to SPC mode, TCSPC imposes a TDC per pixel, it lacks the possibility of storing multiple photon counts per pixel per time-frame, and it requires a longer readout of all pixel TDC data.

When scaling down the technology node leads to better performance electronics, SPAD intrinsic response becomes the limiting factor in achieving fast and precise gating operation. Indeed, high doping concentrations of advanced CMOS technologies worsen SPAD performance. While timing jitter is beneficially decreased with higher bias voltages, power consumption also increases due to the higher avalanche current. Considering that PDP and DCR (even if attenuated by the SPAD on/off ratio) also increase with bias voltage, proper trade-offs should be set for each specific Raman application.

Recent progresses in 3D-stacking [84,85,86,87] show the way towards next-generation high-performance SPAD imagers, by solving the trade-off between SPADs and processing electronics optimization, since top and bottom tiers can be implemented in different technologies, one optimized for SPADs and the other for high functionality and low power consumption electronics.

## Figures and Tables

**Table 1:** Comparison of SPAD arrays presented in Section 4, in terms of detection method, gating mode, delay line implementation, and readout mode.

	**SPC**	**TCSPC**
**Detection method**	128 × 128 SPAD camera [63]	256 × 2 TRFS line sensor [68]
	1024 × 8 SPAD line sensor [66]	16 × 256 TDC-based SPAD line detector [73]
	128 × 1 Multiphase time-gated SPAD line detector [56]	512 × 16 SPAD line sensor with per-pixel histogramming TDC [75]
	256 × 2 TRFS line sensor [68]	128 × 1 time-gated SPAD array [78]
		256 LinoSPAD camera [72] (off-chip)
**Gating mode**	**“Hard-gating”**	**“Soft-gating”**
	128 × 128 SPAD camera [63]	256 × 2 TRFS line sensor [68]
	1024 × 8 SPAD line sensor [66]	
	128 × 1 Multiphase time-gated SPAD line detector [56]	
	16 × 256 TDC-based SPAD line detector [73]	
	512 × 16 SPAD line sensor with per-pixel histogramming TDC [75]	
	128 × 1 time-gated SPAD array [78]	
**Delay line** **implementation**	**Off-chip**	**On-chip**
	128 × 128 SPAD camera [63]	1024 × 8 SPAD line sensor [66] (off-chip coarse and on-chip fine delay line)
	128 × 1 Multiphase time-gated SPAD line detector [56]	512 × 16 SPAD line sensor with per-pixel histogramming TDC [75]
	256 × 2 TRFS line sensor [68]	
	16 × 256 TDC-based SPAD line detector [73]	
**Readout mode**	**Rolling shutter**	**Global shutter**
	128 × 128 SPAD camera [63]	128 × 1 Multiphase time-gated SPAD line detector [56]
		16 × 256 TDC-based SPAD line detector [73]
		the 512 × 16 SPAD line sensor with per-pixel histogramming TDC [75]
		128 × 1 time-gated SPAD array [78]
		256 × 2 TRFS line Sensor [68]
